# Supplementary material for: The Implications of Climate Change on Health among Vulnerable Populations in South Africa: A Systematic Review
Source: Int J Environ Res Public Health. 2023 Feb 15;20(4):3425. doi: 10.3390/ijerph20043425 (PMC9959885; doi:10.3390/ijerph20043425)
Supplement: Supplementary file 1 [file ijerph-20-03425-s001.zip › Supplementary File S2. Quality assessment.pdf]

Quality Assessment

Author/Year	Title	Method		Level of Quality		Include	
			High(H)	Moderate (M)	Low(L)	Yes(Y)	No(N)
Averchenkova et al.,2019	Governance of climate change policy: A case study of South Africa	Report/grey lit	H			Y	
Barnwell,2021	The psychological and mental health consequences of climate change in South Africa	Report		M		Y	
Chersich et al.,2018	Impacts of Climate Change on Health and Wellbeing in South Africa	Systematic Review	H			Y	
Chersich& Wright,2019	Climate change adaptation in South Africa: a case study on the role of the health sector	Systematic Review	H			Y	
Department of Forestry, Fisheries and the Environment, 2020	National climate change adaptation strategy	National Report		M		Y	
Djoudi et al.,2016	Beyond dichotomies: Gender and intersecting inequalities in climate change studies	Review	H			Y	
Islam& Winkel, 2017	Climate Change and Social Inequality	Analytical		M		Y	

Author/Year	Title	Method		Level of Quality		Include	
			High(H)	Moderate (M)	Low(L)	Yes(Y)	No(N)
Mickie, 2020	The Inequality of Climate Change A within-case study on the impact of drought on violence against women in South Africa	Qualitative		M		Y	
Mailula, 2020	Rural Dwellers, Already Prone to Geopolitical and Economic Marginalization, are Predominantly Affected by Climate Change in South Africa	South Africa News Brief and Action Alert			L	Y	
Ngumbela et al.,2020	Local worlds: Vulnerability and food insecurity in the Eastern Cape province of South Africa	Mixed	H			Y	
Petrie et al., 2019	Multi-level climate governance in South Africa Catalysing finance for local climate action	Report	H			Y	
Republic of South Africa, 2019a	Draft national climate change adaptation strategy	Report			L	Y	
Versey,2021	Missing Pieces in the Discussion on Climate Change and Risk: Intersectionality and Compounded Vulnerability	Report		M		Y	
Venter et al.,2020	Green Apartheid: Urban green infrastructure remains unequally	Quantitative	H			Y	

Author/Year	Title	Method		Level of Quality		Include	
			High(H)	Moderate (M)	Low(L)	Yes(Y)	No(N)
	distributed across income and race geographies in South Africa						
Udo, 2020	Gender and climate change adaptation in south africa: A case study of vulnerability and adaptation experiences of local black African women to flood impacts within the Ethekwini Metropolitan municipality	Case study		M		Y	
Wolpe &Reddy,2015	The contribution of low-carbon cities to South Africa's greenhouse gas emissions reduction goals Briefing on urban energy use and greenhouse gas emissions	Report		M		Y	
Ziervogel et al., 2014	Climate change impacts and adaptation in South Africa	Review		M		Y	
Nkosi et al., 2015	Chronic respiratory disease among the elderly in South Africa: any association with proximity to mine dumps?	Cross-sectional study	H			Y	
Republic of South Africa, 2019b	National Development Plan Vision 2030: Our future, make it work	Report		M		Y	
Republic of South Africa, 2020	How unequal is South Africa?	Report		M		Y	

Author/Year	Title	Method		Level of Quality		Include	
			High(H)	Moderate (M)	Low(L)	Yes(Y)	No(N)
Mokoena & Dolan,2020	Climate Change's Disproportionate Impact on Women: Agricultural Workers in South Africa	Online article			L	Y	
Shayegh &Dasgupta, 2022	Climate change, labour availability and the future of gender inequality in South Africa	Survey	H			Y	
Tadese, 2021	Environmental Racism in South Africa: Assessing the Impacts of Durban South Industrial Basin	Working Paper		M		Y	
Tibesigwa & Visser, 2016	Assessing gender inequality in food security among small-holder farm households in urban and rural South Africa			M		Y	
TOTAL			9	12	3	24	0
