# Supplementary material for: The Implications of Climate Change on Health among Vulnerable Populations in South Africa: A Systematic Review
Source: Int J Environ Res Public Health. 2023 Feb 15;20(4):3425. doi: 10.3390/ijerph20043425 (PMC9959885; doi:10.3390/ijerph20043425)
Supplement: Supplementary file 1 [file ijerph-20-03425-s001.zip › Supplementary Table S1. Included studies for the review.pdf]

Table of included studies for the review

No	Document Type	Publication Year	Author(s)	Study Title	Data Extraction	Categories
1	Journal	2015	Nkosi	Chronic respiratory disease among the elderly in South Africa: any association with proximity to mine dumps?	• The production of the toxic gases and dust in the air in mining dumps has increased the prevalence of respiratory diseases among lower-income populations.<br>• The prevalence of asthma (17.3 %), chronic bronchitis (13.4 %), chronic cough (26.6%), emphysema (5.6%), pneumonia (17.1%), and wheeze (24.7%) respectively in the exposed communities.	Geographical location
2	Expert Report	2021	Brandwell	The psychological and mental health consequences of climate change in South Africa	• Access to domestic supply networks and essential services for black population are denied whereas white people can have full access to those services.<br>• Lack of other social determinants such as access to inadequate healthcare, emergency services and adequate schooling makes these population groups more vulnerable to the climate crisis.	Accessibility of resources
3	Journal	2020	Ngumbela et al.	Vulnerability and food insecurity in the Eastern Cape province of South Africa	• Food insecurity caused by climate change is a mediated factor that forced migration among women in South Africa. Climate-related female migrants in South Africa have relatively few alternative livelihood options, apart from sex workers.<br>• Working-class people and informal settlements are experiencing devasting impacts including food security due to crop failure and higher food prices, heat related problems with higher energy prices, water shortages and displacement.<br>• Colored and disadvantaged vulnerable groups are still experiencing the ongoing and intersecting impacts of	Socioeconomic Factors

No	Document Type	Publication Year	Author(s)	Study Title	Data Extraction	Categories
					climate change since they experienced group-based discrimination and are excluded from a variety of contexts.	
4	Government Report	2020	RSA	How unequal is South Africa?	• Economic inequality in South Africa is closely connected to the dimensions of class, race, and gender. Female workers receive 30% wages less than their male counterparts and male employees are more likely to be hired and have higher-paying jobs.<br>• 75% of the population is still living below the poverty line with a higher unemployment rate.<br>Furthermore, racialized inequality exists in the labor market.	

No	Document Type	Publication Year	Author(s)	Study Title	Data Extraction	Categories
5	Policy Report	2019	Averchenkova et al.	Governance of climate change policy: A case study of South Africa	• Lack of data about climate change impacts on the vulnerable population create inaccuracy in estimating the adaption cost and in identifying feasible finance solutions and create a significant barrier to undertake specific implementation.<br>• Lack of the credible comprehensive data on the current climate change in South Africa create challenges for adaptation and undermine the legitimacy of policy.<br>• The policy formulation and implementation in South Africa are difficult due to the complex nature of climate responsibility of governments, which includes poor integration of adaptation policies across jurisdictional levels, poor involvement of stakeholders and poor integration knowledge.	Limitation of the strategy<br><br>Lack of human and financial resources

No	Document Type	Publication Year	Author(s)	Study Title	Data Extraction	Categories
6	Working Paper	2020	Udo	Gender and climate change adaptation in South Africa: a case study of vulnerability and adaptation experiences of local black African women to flood impacts within the eThekwini Metropolitan Municipality	• South African governance today appears to practice a bottom-up approach, with the three levels of government (national, provincial, and local) having the authority to impose their own rules and regulations for climate change adaptation while cooperating and collaborating to work together.	The Strategy Overview
7	Technical Report	2015	Wolpe & Reddy	The contribution of low-carbon cities to South Africa's greenhouse gas emission reduction goals. Briefing on urban energy use and greenhouse gas emissions	• The National Climate Change Response White Paper mentions that bottom-up approach is important for climate adaptation and invites for a coordinated approach from the three levels of government, however, implementations of those policies in their local context are not mandatory.	The Strategy Overview
8	Journal	2019	Petrie et al.	Multilevel Climate Governance in South Africa: Catalyzing Finance for Local Climate Action	• Municipalities are responsible to provide accountable government, whereas the provincial governments are responsible for ensuring that municipal governments follow their regulations and standards, and only intervene when serious concerns about the climate governance arise.	The Strategy Overview

No	Document Type	Publication Year	Author(s)	Study Title	Data Extraction	Categories
9	Policy Report	2019a	RSA	Draft National Climate Change Adaptation Strategy	• In 2016, South Africa has signed the Paris Agreement. Moreover, in line with the commitment to the Paris Agreement, South Africa has developed the National Climate Change Adaptation Strategy to introduce adaptation measures of the climate change effects while limiting the temperature and achieving the stabilization of greenhouse gas emissions.	The evolution of NCCAS
10	Policy Report	2020	DEA	National Climate Change Adaptation Strategy	• The strategy framework aims to identify the underlying causes of vulnerability and gender inequality, and their related risks and implications to individuals within communities and municipalities. NCCS proposed that these climate services to be developed in key vulnerable sectors with the provisional and local municipal governments participation, and implement in areas where risks have been identified, as well as among vulnerable population groups such as rural farmers and coastal communities.<br>• South Africa is making significant progress, at both research and applied levels with the understanding of the climate change impacts, and the adaptation strategy has been well framed and approved to implement and make responses.<br>• South Africa lacks a strong data-based system for extensive climate data, which makes the framework difficult to obtain national climate change and generate through the development framework.	Strength and limitations of the strategy

No	Document Type	Publication Year	Author(s)	Study Title	Data Extraction	Categories
11	Policy Report	2019b	RSA	National Development Plan Vision 2030: Our future, make it work	• National Climate Change Adaptation Strategy recognizes the country's structures and dimensions of various remaining inequalities, resulted from the policies of apartheid.<br>• Evidence from earlier collaborations demonstrates a decrease in the prevalence of infectious diseases such as malaria, diarrhea, and pneumonia, as well as their associated mortality rates.	Strength of the strategy
12	Journal	2019	Chersich & Wright	Climate change adaptation in South Africa: a case study on the role of the health sector	• Although the country has South African Risk and Vulnerability Atlas (SARVA) system which the health risk, driver, exposures, vulnerabilities can be analyzed, the lack of strong scientific climate database and health data system hindered to prepare specific actions on forthcoming climate health impacts.<br>• The lack of sufficient human resources in South Africa's health system has hampered efforts to prepare the health system resilient to extreme weather events and forthcoming climate-related infectious illnesses. Lack of human resources frequently creates considerable limitation in gathering data on climate change-related health impacts, which make it difficult to do health impact assessment.<br>• Lack of financial resources often leads to poor financial aid provision to research institutions, researchers, and practitioners. Of more concern, there are very less research outputs related to health and climate change approximately only 3% of the publications mentions about health impacts out of 600 Publications in 2015.	Limitations of the strategy
13	Journal	2014	Ziervogel et al.,	Climate change impacts and adaptation in South Africa	• Many stakeholders in South Africa, including the government, civil society, researchers, practitioners, and the private sector, have poor relationships when it comes to generating climate change solutions.	Limitations of the strategy

No	Document Type	Publication Year	Author(s)	Study Title	Data Extraction	Categories
					• South Africa has very limited expertise in tackling climate related issues, and key several departments are understaffed.<br>• Existing social inequality caused by poverty is a driving force behind rising multidimensional inequality and climate change inequality.	
14	Journal	2021	Versey, H. S.	Missing pieces in the discussion on climate change and risk: Intersectionality and compounded vulnerability	• Existing social inequality caused by poverty is a driving force behind rising multidimensional inequality and climate change inequality. Climate change's impact on health cannot be separated from the social, economic, cultural, and historical factors that influence health, and it cannot be explained without considering the interconnectedness of such factors and the ways in which they are linked.	Socioeconomic factors
15	Government Report (Journal)	2020	Venter et al.	Green Apartheid: Urban green infrastructure remains unequally distributed across income and race geographies in South Africa	• In South Africa, disparities in access to healthcare, resources, income, morbidity, and mortality persist in vulnerable populations, particularly along economic, racial and gender lines.<br>• It is a country where its racial minority group, White Europeans, are the wealthiest group in the country, while 86% of the remaining population, mainly Black South Africans experiences several inequalities. Although the apartheid regime was abolished in 1994, these disadvantaged groups, such as Black Africans, including Colored, Asians and mixed-race people continue to face systemic inequalities from years of oppression.<br>• The inequality in green spaces contributes adversely to climate-related risks such as air pollution, climate exposure and heat waves. While these green spaces serve as an important social and cultural factor, in South Africa, they	Accessibility of recourses<br><br>Multidimensional inequality

No	Document Type	Publication Year	Author(s)	Study Title	Data Extraction	Categories
					also serve as a source of income for poorer communities through its agricultural use.	
16	Journal	2018	Chersich et al.	Impacts of climate change on health and wellbeing in South Africa	• In 2015, 4% of deaths are contributed by air pollution, and the country is experiencing upward trends in temperature. Furthermore, food security is jeopardized, with crop yields expected to fall across the country, accompanied by livestock failure.<br>• Climate-related female migrants in South Africa have relatively few alternative livelihood options, apart from sex workers, due to a lack of education and lack of jobs in the country. They are more likely to engage in sexual work, then that poses an increased rate of HIV transmission among women. Besides, they often experience gender-based violence.<br>• Women carry additional burdens, such as travelling further to collect water for agricultural use and daily use during droughts in South Africa. These additional burdens also contribute to gender-based violence among girls and women in the country. The rate of gender-based violence and crime against women in South Africa have increased from 0.9%, accelerated to (8.2%) and (9.6%) in 2015 and 2016.	Level of climate risk<br><br>Multidimensional inequality
17	Journal	2016	Djoudi et al.	Beyond dichotomies: Gender and intersecting inequalities in climate change studies	• In South Africa, combined with Apartheid legacies and its ongoing inequitable distribution of natural resources and power imbalance contributes to environmental racialization, where black and people of colored continue to live in the most climate risky areas.<br>• Disadvantaged racial groups are placed to work in the climate destruction process such as mines, the coal-fired	Multidimensional inequality

No	Document Type	Publication Year	Author(s)	Study Title	Data Extraction	Categories
					power stations, steel mills, incinerators, and waste sites or polluting industries, where they are paid low salaries.	
18	Working paper	2020	Mackie	The Inequality of Climate Change: A within-case study on the impact of drought on violence against women in South Africa	• Due to unequal access to land among women in South Africa, they are structurally disadvantage in term of agricultural production. Conjoined with this inequality, the climate change in South Africa caused crop failures and decline in agricultural production. It resulted in a decrease in on-farm employment, leaving women farmers with a loss of income in addition to rising food prices.<br>• Climate related migration creates risks of experiencing human trafficking and sexual exploitation among women. South Africans account for 62 % of trafficking victims, with most of them being women, commonly exploited in sex labor and forced labor while also subjected to violence.	Multidimensional inequality
19	Issues Paper	2020	Mailula	Rural Dwellers, Already Prone to Geopolitical and Economic Marginalization, are Predominantly Affected by Climate Change in South Africa	• South Africa's vulnerability to climate change is significantly increased by economic inequality, poverty, and reliance on coal-fired power generation, all of which exacerbate existing inequalities.	Socioeconomic factors
20	Journal	2020	Mokoena & Dolan	Climate Change's Disproportionate Impact on Women: Agricultural Workers in South Africa	• Women in South Africa suffer the most from the effects of the climate crisis due to their economic marginalization, political exclusion, and distinct labor responsibilities.<br>• When combined with climate change and other social determinants, contributed to decreased financial status, decreased capacity to provide for their children and families, increased climate-related gender-based violence and food insecurity.	Multidimensional inequality

No	Document Type	Publication Year	Author(s)	Study Title	Data Extraction	Categories
					• Although climate change affects everyone, they differ disproportionately depending on identity intersections such as race, class, sexual orientation, and gender.	
21	Journal	2022	Shayegh & Dasgupta	Climate change, labour availability and the future of gender inequality in South Africa	• 60-80% of women in South Africa are involved in the agricultural sector for their livelihood and those of their families depend on their supply and the farm's productivity, both of which are highly vulnerable to negative impacts of climate change.	Socioeconomic Factors
22	Journal	2021	Tadese	Environmental Racism in South Africa: Assessing the Impacts of Durban South Industrial Basin	• Poor and black people have been forcibly relocated to climate-risk areas, such as coastal regions and urban industrial communities with poor air quality, where they are exposed to high levels of hazardous pollution.<br>• In Gauteng province shows that 1.6 million of black Africans are residing in mining dumps in the Gauteng province that are contaminated with uranium and hazardous heavy metals like arsenic, aluminum, manganese, and mercury.	Geographical Location<br><br>Multidimensional inequality
23	Working Paper	2017	Islam & Winkel	Climate change and social inequality	• Intersectionality primarily focuses on a variety of multi-level interrelationships among social locations, forces, factors, and power structures that shape and influence human life. This approach can be used when assessing different types of inequalities caused by climate change.	Multidimensional inequality
24	Journal	2016	Tibesigwa & Visser	Assessing gender inequality in food security among small-holder farm households in urban and rural South Africa	• Male-headed households own larger plots of land and have access to agricultural credits compared to female-headed households, which limits the poor women's participation in agricultural activities and places them at an additional disadvantage, such as engaging in multiple part-time activities to make additional income for their households.	Multidimensional inequality

No	Document Type	Publication Year	Author(s)	Study Title	Data Extraction	Categories
					• 53.1% of female-headed households experience chronic food insecurity and income uncertainty, while 44.12 % of male-headed households are food insecure.	
